# Minimally invasive versus open esophagectomy after neoadjuvant therapy for esophageal cancer: a meta-analysis

**DOI:** 10.1186/s13019-023-02180-x

**Published:** 2023-03-21

**Authors:** Zixian Jin, Kanghao Zhu, Jiajing Sun, Jian Zhang, Bo Zhang

**Affiliations:** 1 Key Laboratory of Minimally Invasive Techniques and Rapid Rehabilitation of Digestive System Tumor of Zhejiang Province, Taizhou Hospital of Zhejiang Province Affiliated to Wenzhou Medical University, Linhai, 317000 China; 2 Department of Thoracic Surgery, Taizhou Hospital of Zhejiang Province Affiliated to Wenzhou Medical University, Linhai, 317000 China; 3 Department of Thoracic Surgery, Taizhou Hospital of Zhejiang Province, Zhejiang University, Linhai, 317000 China

**Keywords:** Neoadjuvant, Minimally invasive, Open, Survival, Safety

## Abstract

**Objectives:**

Neoadjuvant therapy and minimally invasive esophagectomy (MIE) are widely used in the comprehensive treatment of esophageal cancer. This study aimed to investigate the advantages of MIE for esophageal cancer after neoadjuvant therapy.

**Methods:**

Published clinical studies were reviewed and survival data and safety data were extracted. We compared the long-term survival and safety of MIE versus open esophagectomy after neoadjuvant surgery in a series of meta-analyses.

**Results:**

6 retrospective studies were included. Overall, MIE could significantly improve the overall survival of patients with esophageal cancer after neoadjuvant therapy compared with open esophagectomy [hazard ratio (HR) = 0.86, 95% confidence interval (CI) (0.75, 0.98)]. Compared with open esophagectomy, MIE could significantly reduce intraoperative blood loss and operative time [mean difference (MD) = −40.28.78, 95% CI (− 62.98, − 17.58); MD = −28.78, 95% CI (− 42.48, − 15.07), respectively]. There was no significant difference in 30-day and 90-day mortality between MIE and open esophagectomy [odds ratio (OR) = 0.42, 95% CI (0.09, 2.01); OR 0.80, 95% CI (0.25, 2.60), respectively]. MIE could not significantly reduce the incidence of anastomotic leakage, recurrent laryngeal nerve palsy and chylothorax [OR 0.70, 95% CI (0.37, 1.32); OR 1.43, 95% CI (0.33, 6.25); HR = 1.79, 95% CI (0.67, 4.75), respectively], but the incidence of pneumonia was significantly reduced [HR = 0.43, 95% CI (0.22, 0.82)]. In addition, the length of hospital stay and the incidence of total complications were significantly reduced after MIE [MD = −2.61, 95% CI (− 3.10, − 2.12); HR = 0.66, 95% CI (0.45, 0.98), respectively].

**Conclusion:**

MIE after neoadjuvant therapy is effective and safe. Compared with open esophagectomy, MIE can improve the long-term survival and reduce the incidence of postoperative complications of esophageal cancer patients.

**Supplementary Information:**

The online version contains supplementary material available at 10.1186/s13019-023-02180-x.

## Introduction

The incidence of esophageal cancer is increasing year by year, causing a great medical burden [1]. Surgery is the basis of the treatment of esophageal cancer. Minimally invasive surgery has been widely used in the treatment of esophageal cancer. Compared with open esophagectomy, minimally invasive esophagectomy (MIE) reduces the incidence of postoperative complications and is no less effective than open esophagectomy in terms of long-term survival [2]. Neoadjuvant therapy is a commonly used method for preoperative disease control of esophageal cancer, and increases the radical resection rate and postoperative survival time [3, 4].

However, for patients with esophageal cancer after neoadjuvant therapy, the increased difficulty of surgery prompts surgeons to adopt open esophagectomy in many cases [5]. It is still controversial whether to choose open esophagectomy or MIE after neoadjuvant therapy. For example, some studies showed that the incidence of postoperative complications such as esophageal anastomotic leakage would increase after neoadjuvant therapy, but other studies held opposing views [6–9]. After neoadjuvant therapy, surgical selection of esophageal cancer needs further study.

In this meta-analysis, we will review previous studies comparing MIE versus open esophagectomy after neoadjuvant therapy for esophageal cancer, to explore the advantages of MIE after neoadjuvant therapy in terms of long-term survival and safety, and further recommend surgical method.

## Material and methods

### Search strategy

**Fig. 1:**
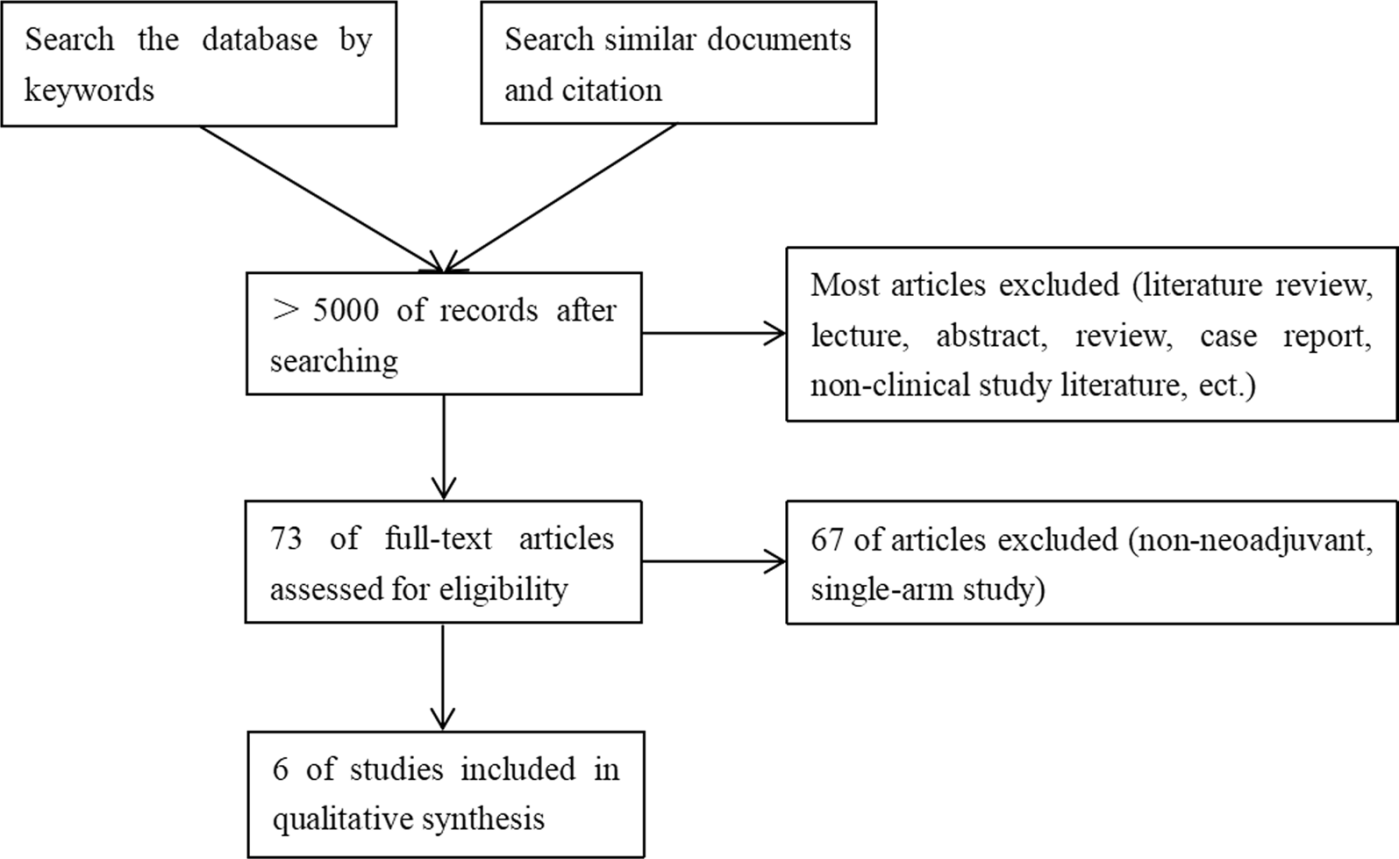
Detailed flowchart of inclusion and exclusion criteria for the literature search

Based on the guidelines for systematic reviews and meta-analysis (PRISMA), literature searches were conducted via Pubmed, Embase, Web of Science and the Cochrane Central Register of Controlled Trials (Fig. 1) [10], from January 1, 2000, to May 31, 2022. The key words were: ((neoadjuvant) OR (preoperative)) AND ((oesophagectomy) OR (thoracoscopy) OR (laparoscopy) OR (minimally invasive)) AND ((esophageal) OR (oesophagus)). We slso searched references of relevant published studies and review articles to supplement the insufficient of keyword retrieval.

### Inclusion and exclusion criteria

Inclusion criteria: (1) Type of study: published clinical study; (2) Subjects: esophageal cancer diagnosed by histopathology; (3) Intervention measures: All patients received neoadjuvant therapy before surgery. The study group (MIE group) underwent thoracoscopic combined with laparoscopic (or laparotomy) esophagectomy, while the control group (Open group) underwent traditional open esophagectomy. Exclusion criteria: (1) Literature review, lecture, abstract, review, case report; (2) Non-clinical study literature, and single-arm study literature; (3) Subjects were previously associated with any malignant tumors; (4) Literature not written in the English language. For studies with the same set of data published repeatedly, the literature with the most complete data will be selected.

### Literature quality assessment

Two researchers independently read the literature titles and abstracts. After excluding the studies that obviously did not meet the inclusion criteria, the full text of the studies that might meet the inclusion criteria was read to determine whether they met the inclusion criteria. Then cross-check was carried out, and if there was any disagreement, a third researcher would decide whether to include literature or not. The quality of the included literature was assessed according to the Newcastle–Ottawa Scale to determine whether there were quality defects [11].

### Data extraction and statistical analysis

The number of patients, intraoperative blood loss, number of lymph node dissection, operation time, postoperative hospital stay, mortality within 30 days and 90 days after surgery, postoperative complications, postoperative survival and other information were extracted from the literature. Meta-analyses were performed in Revman software. Random effects models were used to combine effect sizes regardless of heterogeneity. Odds ratio (OR) was used to compare categorical variables, mean difference (MD) was used to compare continuous variables, and hazard ratio (HR) was used to compare time-survival variables. The significance of the results was expressed as a 95% confidence interval (CI). I^2^ quantifies inter-study heterogeneity, I^2^ ≥ 50% and P ≤ 0.05 indicate heterogeneity. If the heterogeneity is too large, sensitivity analysis was performed to find the source of heterogeneity. Publication bias was represented by funnel plot. Our primary endpoint was overall survival (OS).

## Results

### Literature retrieval results and quality assessment

 > 5000 articles were obtained by keyword retrieval. Excluding literature review, lectures, abstracts, reviews, case reports and non-clinical studies, 73 articles were left. Excluding 67 non-neoadjuvant or single-arm studies, 6 articles remained with a total of 1826 patients. The 6 articles were retrospective studies, among which two were propensity score matching studies [12–17]. The literature were evaluated according to the Newcastle–Ottawa Scale and all literature were of medium to high quality (7–8 score, Additional file 1: Table S1). Therefore, a total of 6 articles were included in the study (Table 1).

**Table 1:** Specific characteristics of 6 studies

Study	Country	Study type	Histology	Adjuvant treatment	Surgical technique	N1	N2	OS	
								HR	95% CI
Chen 2021	China	Single-center retrospective	SCC	NCRT/NCT	Mckeown	120	75	0.61	0.38–0.96
Chen 2022	China	Multi-center PSM	SCC	NCRT	Mckeown/Ivor Lewis	706	353	0.87	0.74–1.02
Hamai 2021	Japan	Single-center retrospective	SCC	NCRT/NCT	Mckeown	68	65	0.83	0.50–1.37
Merritt 2021	USA	Single-center PSM	SCC/AC	NCRT	Ivor Lewis	142	68	0.95	0.59–1.53
Tang 2018	China	Single-center retrospective	SCC	NCT	Mckeown/Ivor Lewis	42	57	1.35	0.35–5.17
Tapias 2016	USA	Single-center retrospective	SCC/AC	NCRT/NCT	Ivor Lewis	56	74	1.07	0.61–1.87

*SCC* squamous cell carcinoma, *AC* adenocarcinoma, *NCRT* neoadjuvant chemoradiotherapy, *NCT* neoadjuvant chemotherapy, *PSM* propensity score matching, *OS* overall survival, *HR* hazard ratio, *CI* confidence interval, *N* number

### Overall survival

**Fig. 2:**
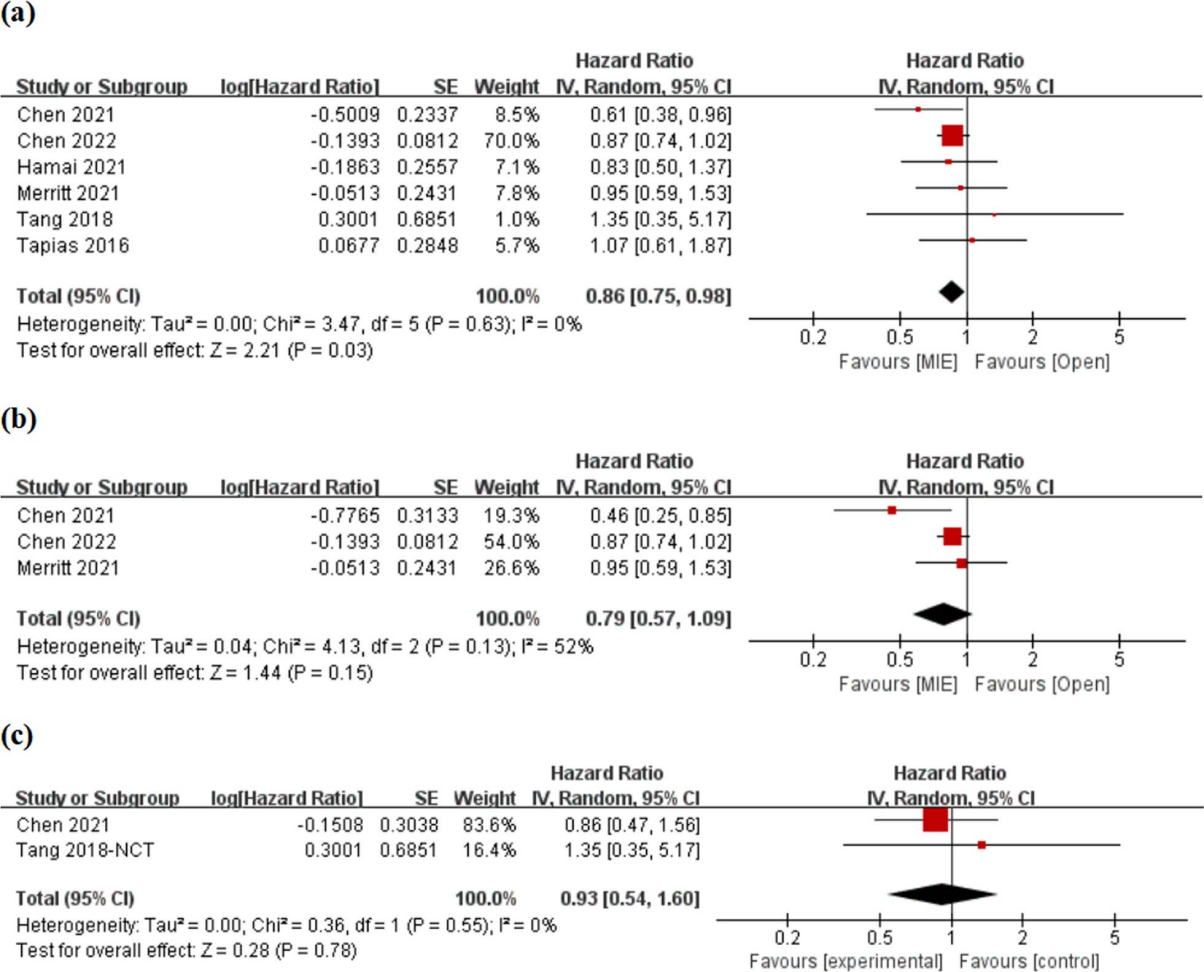
Meta-analyses of overall survival. **a** Neoadjuvant therapy; **b** neoadjuvant chemoradiotherapy; **c** neoadjuvant chemotherapy

All 6 studies provided survival data. Meta-analysis showed that the long-term survival risk of MIE was significantly lower than that of open esophagectomy after neoadjuvant therapy [HR = 0.86, 95% CI (0.75, 0.98)] (Fig. 2a). Heterogeneity test indicated that there was no heterogeneity between studies, I^2^ = 52%, P = 0.63. In addition, subgroup analysis was performed on surgery after neoadjuvant chemoradiotherapy or neoadjuvant chemotherapy. Compared with open esophagectomy, MIE reduced overall survival risk, but the difference was not statistically significant [HR = 0.79, 95% CI (0.57, 1.09); HR = 0.93, 95% CI (0.54, 1.60), respectively] (Fig. 2b, c). Inter-study existed moderate heterogeneity in the subgroup of neoadjuvant chemoradiotherapy (I^2^ = 52%, P = 0.13).

2 studies did a propensity score analysis (Chen 2022 and Merritt 2021), while 4 studies did not. The results of the meta-analysis were similar with or without propensity score analysis [HR = 0.88, 95% CI 0.75–1.02; HR = 0.80, 95% 0.60–1.07, respectively], and there was no significant heterogeneity between studies (I^2^ = 0%, I^2^ = 2%, respectively) (Additional file 1: Fig. S1a, b).

### Safety

#### Intraoperative outcomes

**Fig. 3:**
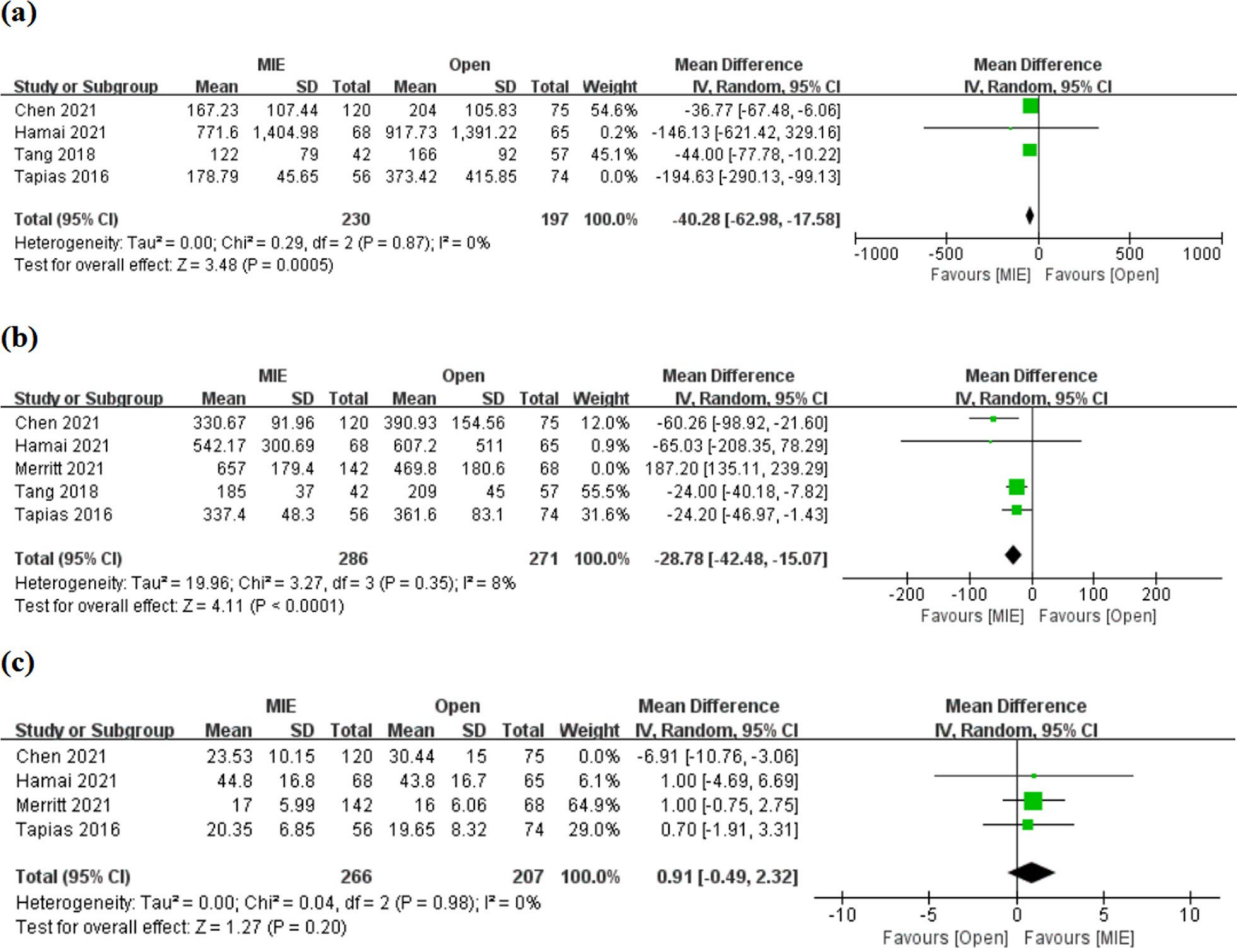
Meta-analyses of intraoperative blood loss (**a**), operative time (**b**), and number of lymph node dissection (**c**) after sensitivity analyses

(1) Intraoperative blood loss: Intraoperative blood loss was reported in 4 studies. Meta-analysis showed that MIE was associated with reduced blood loss [MD = −70.50, 95% CI (− 123.76, − 17.23)], but there was significant heterogeneity between studies, I^2^ = 69%, P = 0.02 (Additional file 1: Fig. S2a). Sensitivity analysis showed that the maximum sensitivity came from the study of Tapias et al. and heterogeneity tended to be stable with I^2^ = 0% after the study was omitted (Fig. 3a; Additional file 1: Fig. S2a). Meta-analysis after sensitivity analysis showed that MIE reduced intraoperative blood loss [MD = −40.28.78, 95% CI (− 62.98, − 17.58)] (Fig. 3a).

(2) Operative time: 5 studies reported operative time. According to analysis, there was no statistically significant difference in operative time between the two interventions [MD = 7.10, 95% CI (− 53.32, 67.52)] (Additional file 1: Fig. S2b). And inter-study existed significant heterogeneity, I^2^ = 94%, P < 0.00001. Sensitivity analysis showed that the maximum sensitivity came from the study of Merritt et al., and heterogeneity tended to be stable with I^2^ = 8% after the study was omitted (Fig. 3b; Additional file 1: Fig. S2b). Meta-analysis after sensitivity analysis showed that MIE reduced operative time [MD = −28.78, 95% CI (− 42.48, − 15.07)] (Fig. 3b).

(3) Number of lymph node dissection: The number of lymph node dissection was reported in 4 studies. Meta-analysis showed no significant difference in lymph node dissection between minimally invasive and open esophagectomy [MD = −0.94, 95% CI (− 4.28, 2.40)] (Additional file 1: Fig. S2c). And inter-study existed significant heterogeneity, I^2^ = 79%, P = 0.003. Sensitivity analysis showed that the maximum sensitivity came from the study of Chen et al., and heterogeneity tended to be stable with I^2^ = 0% after the study was omitted (Fig. 3c; Additional file 1: Fig. S2c). Meta-analysis after the study was omitted showed no significant difference in the number of lymph nodes dissection between the two surgical procedures [MD = 0.91, 95% CI (− 0.49, 2.32)] (Fig. 3c).

#### Postoperative outcomes

**Fig. 4:**
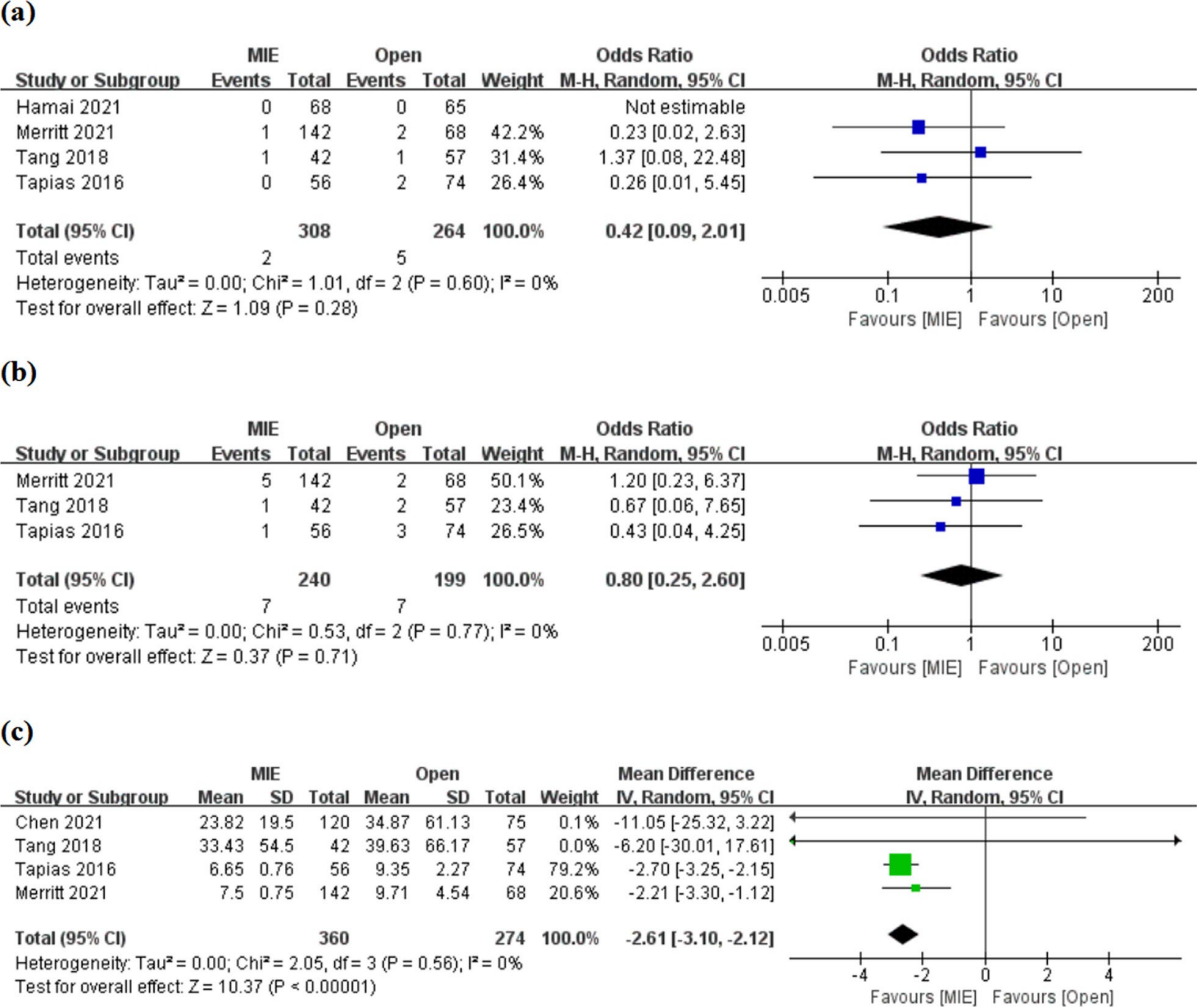
Meta-analyses of 30-day mortality (a), 90-day mortality (**b**) and length of postoperative hospital stay (**c**)

**Fig. 5:**
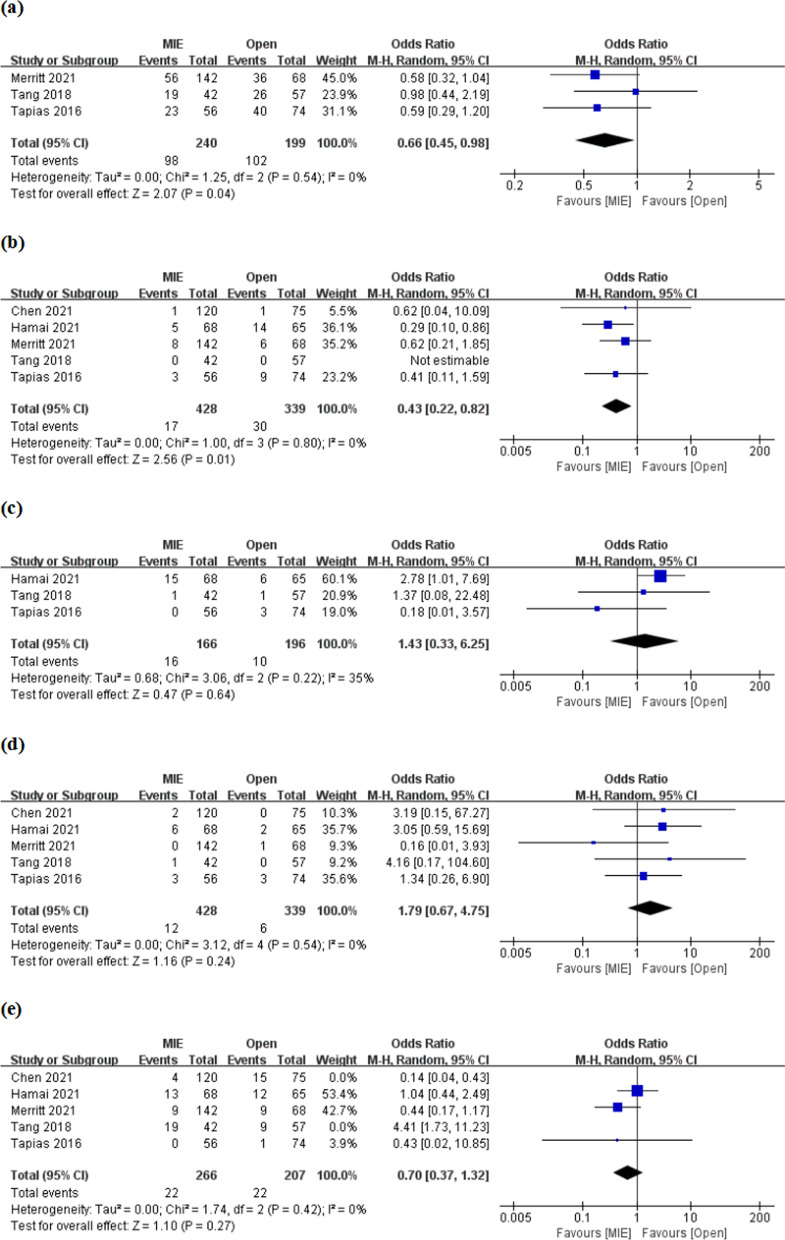
Meta-analyses of incidence of total complications (**a**), especially pneumonia (**b**), recurrent laryngeal nerve palsy (**c**) and chylothorax (**d**); meta-analysis of anastomotic leakage (**e**) after sensitivity analysis

(1) Short-term mortality: 30-day and 90-day mortality were reported in 4 and 3 studies, respectively. There was no significant difference in 30-day or 90-day mortality between minimally invasive and open esophagectomy [OR 0.42, 95% CI (0.09, 2.01); OR 0.80, 95% CI (0.25, 2.60), respectively], and no significant heterogeneity between studies (I^2^ = 0%) (Fig. 4a, b).

(2) Length of postoperative hospital stay: 4 studies reported the length of postoperative hospital stay. Meta-analysis showed reduced length of hospital stay after minimally invasive surgery [MD = −2.61, 95% CI (− 3.10, − 2.12)], with no significant heterogeneity between studies (I^2^ = 0%) (Fig. 4c).

(3) Postoperative complications: 3 studies respectively reported the incidence of total complications and recurrent laryngeal nerve palsy, and 5 studies respectively reported the incidence of pneumonia, chylothorax and anastomotic leakage. Meta-analyses showed that MIE had an advantage in the incidence of total complications, especially pneumonia [HR = 0.66, 95% CI (0.45, 0.98); HR = 0.43, 95% CI (0.22, 0.82), respectively] (Fig. 5a, b). However, there was no significant difference between the two interventions in the incidence of recurrent laryngeal nerve palsy, chylothorax, and anastomotic leakage [OR 1.43, 95% CI (0.33, 6.25); OR 1.79, 95% CI (0.67, 4.75); OR 0.71, 95% CI (0.20, 2.49), respectively] (Fig. 5c, d; Additional file 1: Fig. S3). Significant inter-study heterogeneity was found in the meta-analysis of anastomotic leakage (I^2^ = 83%, P < 0.00001), and sensitivity analysis showed that the largest heterogeneity came from the studies of Chen et al. and Tang et al. (Additional file 1: Fig. S3). However, meta-analysis after the removal of the two studies showed no significant difference in the incidence of anastomotic leakage between the two interventions [OR 0.70, 95% CI (0.37, 1.32)], and heterogeneity tended to stabilize (I^2^ = 0%) (Fig. 5e).

#### Publish bias assessments

The funnel plot is symmetrical, showing no significant publication bias in the meta-analysis of OS (Additional file 1: Fig. S4).

## Discussion

This was a meta-analysis to explore whether MIE is preferred after neoadjuvant therapy for esophageal cancer. Our study showed that MIE after neoadjuvant therapy was effective and safe, with an overall trend of reducing postoperative complications, shortening hospital stay, and improving long-term survival.

Conventional open surgery is more traumatic to patients, so minimally invasive techniques have been widely used in various types of surgery in recent years. In addition to the smaller incision, a significant advantage of minimally invasive technique is the magnification of the endoscope, which can clearly identify very small structures. As a result, the surgery can be performed with greater precision to preserve nerves and blood vessels. With the promotion and improvement of minimally invasive technology, the efficacy and safety of surgical patients have been significantly improved. Surgery is the basic method for radical treatment of esophageal cancer. Similarly, for esophageal cancer surgery, many studies have confirmed that minimally invasive technology has advantages in long-term survival rate and postoperative complications, so it becomes the first choice in many situation [18–20].

However, for locally advanced esophageal cancer, surgery alone is difficult to significantly improve the postoperative survival of patients. In view of this situation, neoadjuvant therapy and adjuvant therapy have been widely studied as surgical supplements for locally advanced esophageal cancer. The CROSS trial, NEOCRTEC5010 trial, and the JCOG9907 trial have demonstrated that neoadjuvant chemoradiotherapy, chemotherapy can show improved postoperative survival for locally advanced esophageal cancer and have been validated in multiple meta-analyses [21–26]. However, after neoadjuvant therapy, it is possible to increase the risk of surgery due to the destruction of esophageal tissue and surrounding structures and adhesion in the thorax. This situation makes surgeons prefer open surgery. Many studies have shown that neoadjuvant therapy increases the risk of postoperative pulmonary complications, cardiovascular complications, and anastomotic leakage. But other studies have reported different results [6–9]. Previous meta-analyses have confirmed that neoadjuvant therapy does not significantly increase the incidence of total complications after surgery [25, 26].

Neoadjuvant therapy and minimally invasive technique are excellent treatment methods for esophageal cancer. However, it is still controversial whether minimally invasive or open esophagectomy should be preferred after neoadjuvant therapy. Overall, our study suggested that MIE was recommended as the first choice. MIE was superior to open esophagectomy in long-term survival [HR = 0.86, 95% CI 0.75, 0.98]. In terms of intraoperative conditions, MIE had short operative time and less bleeding than open esophagectomy. The amount of lymph nodes removed is often considered a short-term proxy for long-term outcomes, and this has been demonstrated in multiple studies [27–29]. NCCN guidelines also recommend that at least 15 lymph nodes be dissected for esophageal cancer. In the studies included in our analysis, lymph node dissection met the requirements of NCCN guidelines, and no significant differences were found between the two surgical procedures. In terms of safety, compared with traditional open esophagectomy, the incidence of recurrent laryngeal nerve palsy and anastomotic leakage after MIE did not increase. What's more, MIE had a lower overall complication rate than open esophagectomy, especially for pneumonia. MIE reduced overall complication rates by 34 percent and pneumonia rates by 57 percent. Cardiovascular complications are also a common problem after esophageal cancer surgery. Although cardiovascular complications were not involved in our study, previous meta-analyses had shown that the incidence of postoperative cardiovascular complications after MIE was lower than that after open esophagectomy [30, 31]. In addition, our study also showed that there was no significant difference in short-term postoperative mortality between the two surgical procedures, and that patients who underwent MIE were discharged from the hospital in less time and recovered more quickly. This means that neoadjuvant therapy combined with minimally invasive surgery is effective and safe, in line with the concept of enhanced recovery after surgery [32]. This was the first meta-analysis to compare the survival benefit and safety of minimally invasive versus open esophagectomy after neoadjuvant therapy for esophageal cancer. We hope this study will provide advice for surgeons on surgical options.

This study is a meta-analysis, and there is no way to obtain exact patient data, so it has its own limitation. All the studies included in this study were retrospective studies, and the selection of patients was subject to surgeons' subjective judgment. Therefore, the quality of the literature is difficult to match that of randomized clinical trials. Moreover, we also found high heterogeneity in multiple comparisons. Heterogeneity might be caused by different surgical methods (Mckeown, Ivor Lewis, total minimal invasive and hybrid minimal invasive), and different protocols of neoadjuvant therapy (chemoradiotherapy and chemotherapy). Therefore, we applied sensitivity analysis and random effects model to reduce heterogeneity's interference with the results. For patients with difficulty in MIE, surgeons are more likely to choose open esophagectomy. In the real world, patients undergoing minimally invasive conversion to open surgery is a common phenomenon. In order to avoid additional damage to the organs around the esophagus, surgeons prefer open surgery when the esophagus is found to be closely attached to the surrounding tissues and organs, difficult to be separated under the endoscopy, and when the tumor is huge and impinges on the trachea, lung and other organs, as well as pleural cavity atresia, and so on. Therefore, the conclusion of our study cannot prove that MIE is superior to open esophagectomy under the premise of neoadjuvant therapy, and it can only be recommended to use MIE under the same situation.

## Conclusion

MIE after neoadjuvant therapy is effective and safe. Compared with open esophagectomy, MIE can improve the long-term survival and reduce the incidence of postoperative complications of esophageal cancer patients. MIE is recommended as the first consideration after neoadjuvant therapy. Multicenter randomized clinical trials are needed to confirm this conclusion.

## Supplementary Information

**Additional file 1.** Supplementary explanatory material to the manuscript.

## Data Availability

Availability of data and materials.
