# Supplementary material for: Minimally invasive versus open esophagectomy after neoadjuvant therapy for esophageal cancer: a meta-analysis
Source: J Cardiothorac Surg. 2023 Mar 21;18:90. doi: 10.1186/s13019-023-02180-x (PMC10029261; doi:10.1186/s13019-023-02180-x)
Supplement: Supplementary file 1 — Additional file 1. Supplementary explanatory material to the manuscript. [file 13019_2023_2180_MOESM1_ESM.docx]

**Supplementary Table 1.** Risk of Bias Assessment: Based on the Newcastle-Ottawa Scale

| Study | Selection | | | | Comparability Control for improtant factor | Exposure | | | Scores |
| --- | --- | --- | --- | --- | --- | --- | --- | --- | --- |
|  | Adequate definition of cases | Representat-  iveness of the cases | Selection of controls | Definition of controls |  | Ascertain-  ment of exposure | Same method of ascertainment for cases and controls | Non-response rate |  |
| Chen 2021 | 1 | 1 | 1 | 1 | 0 | 1 | 1 | 1 | 7 |
| Chen 2022* | 1 | 1 | 1 | 1 | 1 | 1 | 1 | 1 | 8 |
| Hamai 2021 | 1 | 1 | 1 | 1 | 0 | 1 | 1 | 1 | 7 |
| Merritt 2021* | 1 | 1 | 1 | 1 | 1 | 1 | 1 | 1 | 8 |
| Tang 2018 | 1 | 1 | 1 | 1 | 0 | 1 | 1 | 1 | 7 |
| Tapias 2016 | 1 | 1 | 1 | 1 | 0 | 1 | 1 | 1 | 7 |

*The data is based on propensity score matching.

**Supplementary Figure 1** Meta-analyses of studies with **(a)** or without **(b)** propensity score analysis.

**(a)**
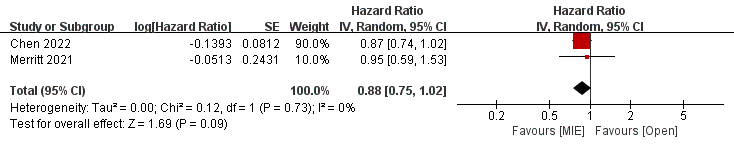


**(b)**
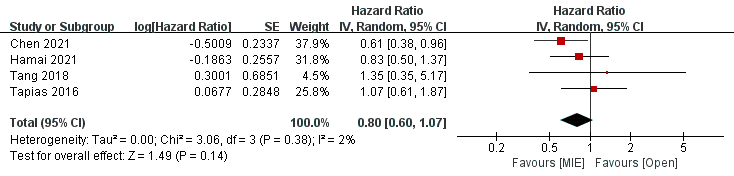


**Supplementary Figure 2** Sensitivity analyses of intraoperative blood loss **(a)**, operative time **(b)**, and number of lymph node dissection **(c)**

**(a)**

**
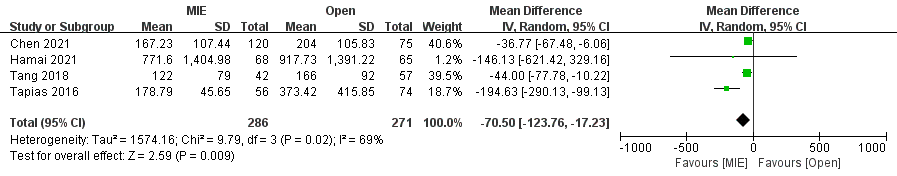
**

**
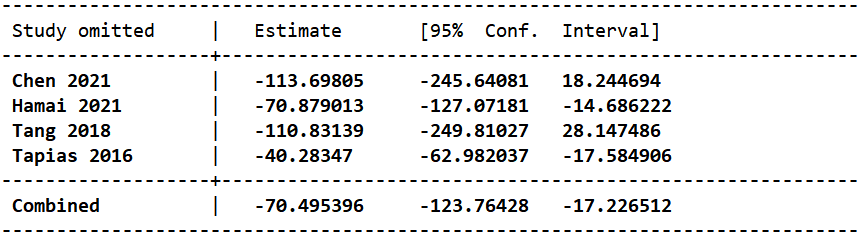

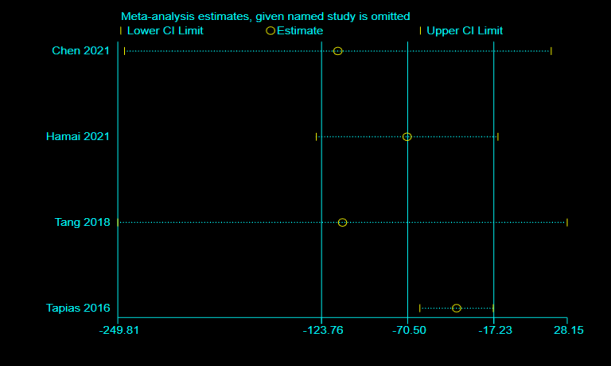
**

**(b)**

**
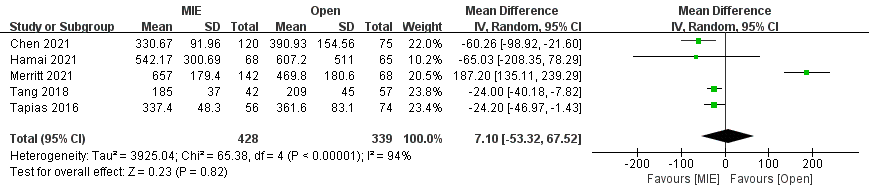
**


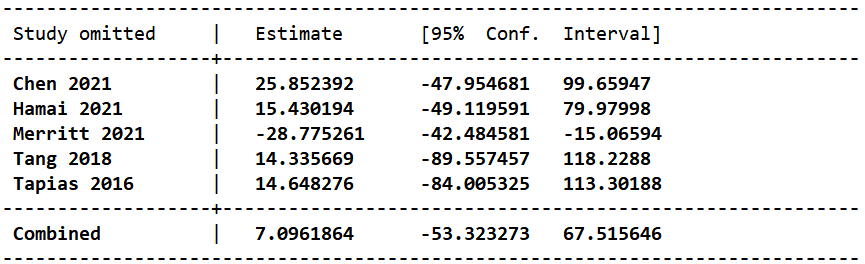

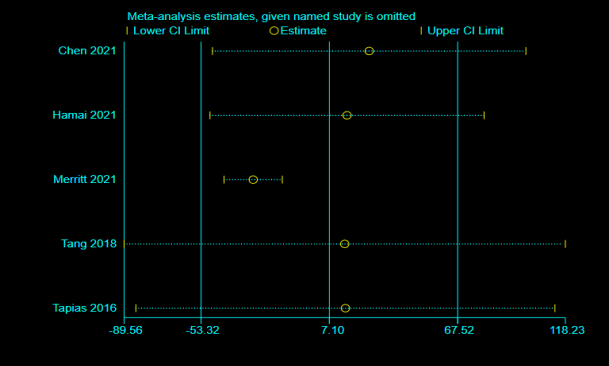


**(c)**


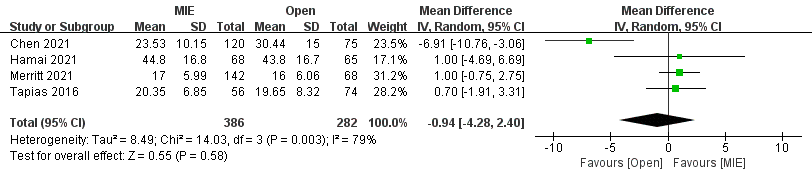


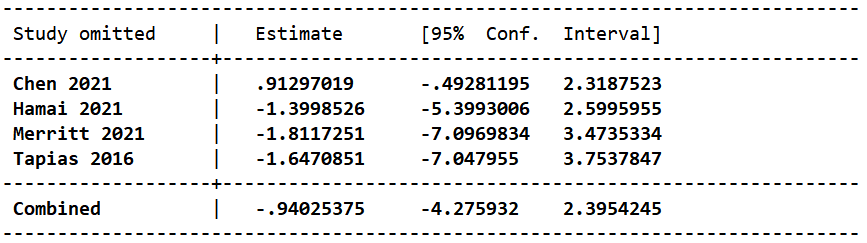

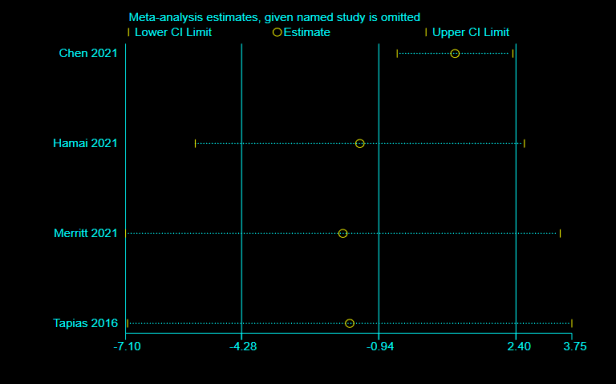


**Supplementary Figure 3** Sensitivity analysis of anastomotic leakage

**
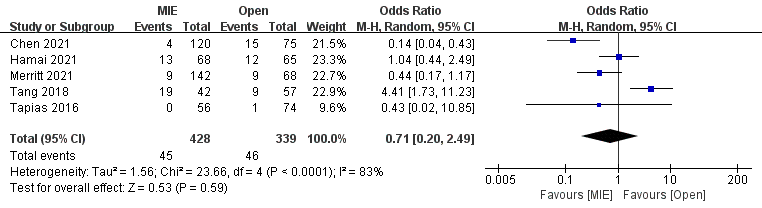
**

**
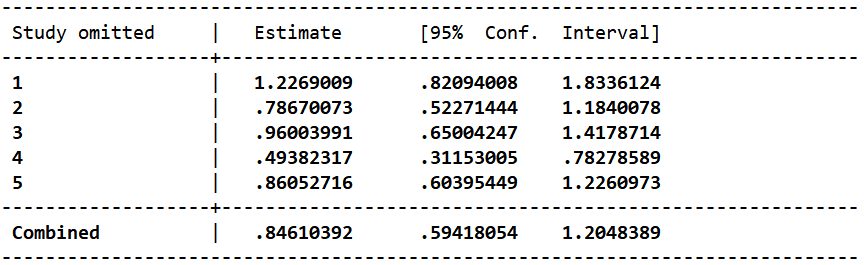

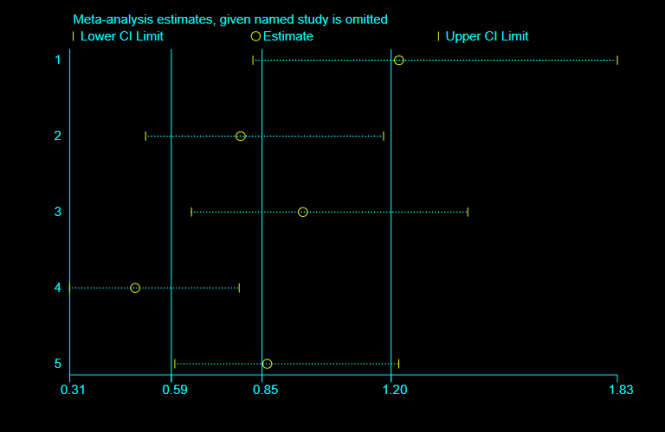
**

**Supplementary Figure 4** Funnel plot of overall survival

**
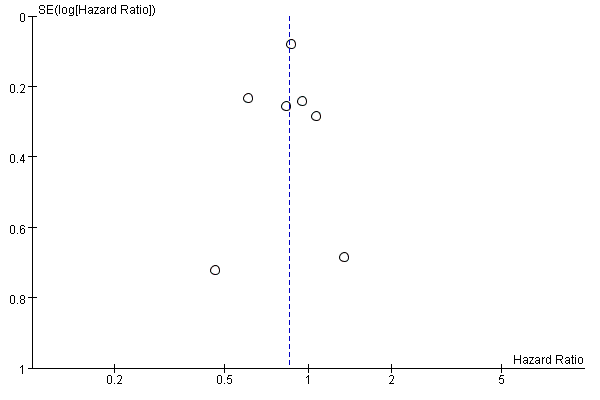
**
